# Difficulties experienced by migrant physicians working in German hospitals: a qualitative interview study

**DOI:** 10.1186/s12960-016-0153-4

**Published:** 2016-09-23

**Authors:** Corinna Klingler, Georg Marckmann

**Affiliations:** Institute of Ethics, History & Theory of Medicine at LMU Munich, Lessingstr. 2, 80336 Munich, Germany

**Keywords:** Qualitative research, Interviews, Migrant physicians, International medical graduates, Professional challenges, Support needs, Germany

## Abstract

**Background:**

With Germany facing a shortage of doctors, hospitals have been increasingly recruiting physicians from abroad. Studies in other countries have shown that migrant physicians experience various difficulties in their work, which might impact the quality of patient care, physician job satisfaction, and, accordingly, retention. The experiences of migrant doctors in Germany have not been systematically studied so far and will likely differ from experiences migrant physicians make in other contexts. A thorough understanding of challenges faced by this group, however, is needed to develop adequate support structures—as required by the WHO Global Code of Practice on the International Recruitment of Health Personnel.

**Methods:**

A qualitative study was conducted to give an overview of the multifaceted difficulties migrant physicians might face in German hospitals. Twenty semi-structured interviews with foreign-born and foreign-trained physicians were conducted in German. Participants were recruited via the State Chambers of Physicians and snowballing based on a maximum variation sampling strategy varying purposefully by source country and medical specialty. The interviews were recorded, transcribed verbatim, and analysed using qualitative content analysis.

**Results:**

Participants described difficulties relating to healthcare institutions, own competencies, and interpersonal interactions. Participants experienced certain legal norms, the regulation of licensure and application for work, and the organization of the hospital environment as inadequate. Most struggled with their lack of setting-specific (language, cultural, clinical, and system) knowledge. Furthermore, behaviour of patients and co-workers was perceived as discriminating or inadequate for other reasons.

**Conclusions:**

This is the first study to describe the broad range of issues migrant physicians experience in Germany. Based on this information, institutional actors should devise support structures to ensure quality of care, physician wellbeing, and retention. For example, training opportunities should be offered where needed to support acquisition of setting-specific knowledge. Discrimination experienced by participants calls for better diversity management as a leadership task in healthcare institutions. Misinformation practices in recruitment could be managed by a voluntary code of ethical conduct. Further research is necessary to identify strategies that adequately address diverging normative positions between migrant health personnel and their patients and colleagues.

## Abstract (de)

**Hintergrund:**

Internationalen Studien zufolge erleben migrierte Ärzte vielfältige Schwierigkeiten bei ihrer Tätigkeit. Dies kann sich negativ auf die Versorgungsqualität, sowie die Arbeitszufriedenheit des migrierten Personals auswirken und dazu führen, dass Ärzte sich gegen die klinische Tätigkeit entscheiden. Um dem Ärztemangel zu begegnen, rekrutieren auch deutsche Krankenhäuser ärztliches Personal zunehmend im Ausland. Die Erfahrungen migrierter Ärzte in Deutschland sind jedoch bisher kaum untersucht worden, unterscheiden sich aber wahrscheinlich von den Erfahrungen, die migrierte Ärzte in anderen nationalen Kontexten machen. Dies erschwert die Entwicklung angemessener Unterstützungsstrukturen für diese Gruppe, wie sie vom WHO Global Code of Practice on the International Recruitment of Health Personnel gefordert werden.

**Methode:**

Eine qualitative Studie wurde durchgeführt, um einen ersten Überblick über die vielfältigen Schwierigkeiten zu erlangen, die migrierte Ärzte in deutschen Krankenhäusern erleben. 20 leitfadengestützte Interviews wurden mit im Ausland geborenen und ausgebildeten Ärzten geführt, die in deutschen Krankenhäusern tätig waren. Teilnehmer wurden mithilfe von vier Landesärztekammern sowie des Schneeballsystems gemäß einer Maximum-Variation-Sampling Strategie rekrutiert, die Herkunftsland und Fachgebiet der Teilnehmer absichtsvoll variierte. Die Interviews wurden aufgenommen, transkribiert und mithilfe der qualitativen Inhaltsanalyse ausgewertet.

**Ergebnisse:**

Die von den Teilnehmern beschriebenen Schwierigkeiten bezogen sich auf die Institutionen des Gesundheitssystems, eigene Kompetenzen und zwischenmenschliche Beziehungen. Teilnehmer erlebten bestimmte rechtliche Normen, den Ablauf des Anerkennungs- sowie des Bewerbungsverfahrens und die Organisation der Arbeitsumgebung als unzulänglich. Viele belastete ihr begrenztes kontextspezifisches Wissen (in Bezug auf Sprache, Kultur, fachliche Erfahrung und Organisationsstruktur). Des Weiteren erlebten Teilnehmer das Verhalten von Patienten und Mitarbeitern als diskriminierend oder anderweitig unangemessen.

**Schlussfolgerungen:**

Dies ist die erste Studie, die systematisch das Spektrum von Problemen erfasst, die immigrierte Ärzte in deutschen Krankenhäusern erleben. Auf der Grundlage dieser Informationen sollten Entscheidungsträger auf den verschiedenen Ebenen des Gesundheitswesens Unterstützungsstrukturen entwickeln und implementieren, um so die Qualität der Versorgung sowie die Arbeitszufriedenheit der Ärzte sicherzustellen. So sollten beispielsweise Weiterbildungsangebote entwickelt werden, die migrierte Ärzte darin unterstützen, kontextspezifisches Wissen zu erwerben – sofern entsprechender Bedarf besteht. Diskriminierung sollte mit einer besseren Führungskultur begegnet werden, die Diversität bei Personal und Patienten wertschätzt. Ein freiwilliger Ethikkodex könnte außerdem fragwürdigen Rekrutierungspraktiken entgegenwirken. Es bedarf weiterer, vor allem normativ-analytischer Reflexion, um angemessene Strategien zum Umgang mit moralischen Wertekonflikten zwischen migrierten Gesundheitsfachkräften und ihren Patienten und Kollegen zu identifizieren.

## Background

### The international context of physician migration

Physician migration has been on the global health agenda since the late 1990s [1] cumulating in the publication of the WHO Global Code of Practice on the International Recruitment of Health Personnel [2]. The Code promotes ethical principles to guide the international recruitment of health personnel in an attempt to address various problematic aspects of international health worker migration. Foremost, the Code engages with the issue of increasing reliance on migrant health workers in many high-income countries and the resulting aggravation of already critical shortages in often poor source countries [3–5]. However, the Code also addresses the inappropriate treatment of migrant physicians within destination countries regarding, for example, limited opportunities for career progression and the lack of orientation programmes to ensure safe practice. For states to be able to conform to the normative principles set out in the Code, a sound evidence base is required [2, 6]. Specifically, a thorough understanding of the difficulties migrant physicians experience in the context of the respective healthcare system is needed if states want to live up to their responsibilities regarding adequate treatment and support. So far, however, only limited research has been conducted on this question as most academic efforts have focused on the so-called brain drain [7].

In the studies that have been conducted—in various national contexts^1^—migrant physicians reported facing several challenges in medical practice. Educational needs and the lack of corresponding support structures have been highlighted by migrant physicians in relation to language [8–18], organization of the healthcare system [10–12, 14, 17, 19–22], sociocultural aspects of communication, and interaction with patients and colleagues, especially regarding patient-centred care [8–24] and clinical knowledge, when facing different disease spectra and treatment methods [10, 20, 22]. Facing insensitivity and rejection or even overt discrimination and racism by patients and colleagues is another important issue [11, 12, 14, 18, 21, 25, 26]. Other struggles mentioned include loss in professional status and recognition often resulting in deskilling [18, 22, 27–29], stressful and lengthy certification processes [10, 17, 25, 26], and difficulties in gaining admission to postgraduate (specialist) training programmes^2^ [19, 28, 29]. Additionally, physicians mentioned difficult working and learning environments in terms of, e.g. work load [26, 28, 29], limited career opportunities, and inadequate information thereof during the recruitment process [28, 29], and difficulties staying in the destination country long-term, as well as feelings of guilt for leaving one’s home country and an already underserved population [21]. The described difficulties can compromise not only the quality of patient care but also physicians’ own wellbeing with regard to mental health and job satisfaction [30, 31], thereby negatively affecting performance [32] and retention of much needed health personnel [28, 33]—providing additional reasons for states to address aforementioned issues in the context of their respective healthcare systems.

### The German situation

Although having a comprehensive account of migrant physicians’ difficulties is a prerequisite for devising adequate support structures, the experiences of migrant doctors in Germany have hardly been investigated. The few studies conducted have mainly addressed routes of, and reasons for, physician migration from and to Germany [34, 35], but to our knowledge, the problems faced by migrant doctors in Germany have never been analysed systematically. One reason might be that Germany was long perceived as only moderately reliant on migrant physicians with a share of around 5 % foreign physicians in 2006 [36]. In 2015, 10 % of practising physicians were foreign (37.878 in absolute numbers) [37], while other countries exhibited rates as high as 58 % (Israel) or 43 % (New Zealand) [38].^3^ Even in the European context, Germany’s reliance on foreign-trained health professionals is low compared to Norway (37 %), Ireland (36 %), the United Kingdom (28 %), and Switzerland (27 %).^4^ However, Germany is one of the countries with the sharpest increase in the share of foreign-educated doctors within the OECD in the last decade [38]. This trend is unlikely to reverse in the near future as Germany faces a shortage of doctors with a predicted gap of up to 106 000 physicians in 2030 (which equates to 23.7 % of full-time positions needed to uphold today’s level of care) [39]. As Germany has not done much on a national level to meet this shortage, for example, increasing medical school capacities [38, 40], it will have to rely on foreign-trained doctors to fill the predicted gap. Accordingly, hospitals in Germany already increasingly recruit doctors from abroad—sometimes with the help of private recruitment agencies [40, 41]. Particularly in rural, presumably less attractive areas in Eastern Germany, hospitals face severe shortages and have become heavily reliant on migrant doctors [35, 42]. Migrant physicians, therefore, play and will play an important role in securing healthcare delivery in Germany; hence, it will be indispensable to better understand how they fare within the German healthcare system and what challenges they face.

Most likely, experiences of migrant doctors in Germany will differ from experiences of foreign-trained doctors in other countries. They cannot easily be translated from one context to another for two reasons: (a) differences in the cultural and organizational set-up between destination countries and (b) differences regarding typical source countries [10].

Obviously, variances in organizational context can lead to variances in experiences of migrant physicians, for example, regarding certification (or licensure) procedures. In Canada, the provincial authorities are responsible for certification with differing rules across provinces [43], which might complicate understanding the requirements for certification. The USA, on the other hand, has a central credentialing body with easily accessible information, but entry requirements are relatively high [44] compared to, for example, regulation in the European Union (EU).^5^ Medical qualifications obtained in the EU are automatically recognized by all member states without additional testing. In the USA, physicians are not offered much support in preparing for the various examinations, e.g., financial loans that would allow them to focus on preparation while being able to meet immediate needs of themselves and their families—support that is, for example, offered in Canada [45]. It will therefore often take a significant amount of time for migrant physicians to become certified in the USA (if at all) resulting in brain waste [46].

Furthermore, the physicians who migrate to Germany come from different source countries than is common in other destination countries. Accordingly, the cultural, system, and linguistic backgrounds of migrant physicians vary between destination countries. Health professionals migrating to Germany come predominantly from the EU, especially from Romania, Greece, and Austria [37]. In this respect, Germany resembles other European countries like Belgium, France, or Italy [36]. Spain and the United Kingdom, on the other hand, have relied more heavily on migrants from non-EU countries [36]. Migration to the United Kingdom, for example, has been based mainly on colonial ties, with India and Pakistan being important source countries [47]. Differences in cultural, system, and linguistic backgrounds influence knowledge and expectations of migrant physicians and will, therefore, likely result in varied challenges and corresponding needs for support and education.

We conducted an interview study to provide a comprehensive overview of the multifaceted difficulties experienced by migrant physicians practising in German hospitals. The results will provide valuable information for developing specific strategies to effectively support this group and to improve the quality of care they provide, their job satisfaction, and their retention.

## Methods

The reporting of methods broadly follows the consolidated criteria for reporting qualitative research [48].

### Study design

As—to our knowledge—no research project has systematically addressed difficulties migrant physicians experience when practising in Germany, an explorative, qualitative study design was deemed appropriate. Furthermore, as we were interested in the perception of the physicians themselves, a descriptive, instead of an interpretative, approach was chosen [49]. Semi-structured interviews were conducted with migrant physicians, defined as doctors born and trained outside of Germany but now working in the German healthcare system. The interview guide was constructed according to Helfferich [50] and based on a non-systematic review of empirical studies published in PubMed, which explore migrant physicians’ experiences in destination countries. In addition to difficulties experienced, it addressed existing and desired support structures (not reported here). The interview guide was discussed with experienced researchers and pilot tested.

### Sampling and recruitment

A maximum variation sampling strategy [51] was chosen to allow capture of a broad spectrum of experienced difficulties. A sample plan [52] was constructed (see Table 1) in which we varied purposefully those characteristics of migrant physicians that we assumed would partially determine variance in experiences: source country (where the physician was born and trained) and speciality.^6^ Accordingly, the sample plan included physicians from the source countries most represented among migrant physicians in Germany. At time of data collection, those were Russia, Greece, Poland, and Romania. Austria, being among the biggest source countries, was excluded as we assumed that due to linguistic, cultural, and organizational proximity, Austrian-trained physicians would have had very similar experiences to German-trained doctors. Additionally, assuming that physicians from non-European countries might face different challenges, the biggest non-European source country (Iran) was included. However, we had to adapt the sample plan and include physicians from other non-European source countries as it proved difficult to find Iranian participants (see Table 2 for final sample). Regarding medical specialties, the two biggest specialties (internal medicine and surgery) as well as psychiatry, for its specific communicative requirements, were included for sample construction. Potential participants were approached through four State Chambers of Physicians. The Chambers sent an information sheet regarding the study to migrant physicians identified via their databases. They were also sent an answer sheet that physicians could fax back to the Chambers indicating their willingness to participate. Additionally, snowballing [53] was used; after the interview, participants were asked whether they knew and were willing to approach potential participants. Five participants were recruited that way.

**Table 1:** Sample plan

	Internal medicine	Psychiatry	Surgery
Romania	≥1	≥1	≥1
Poland	≥1	≥1	≥1
Russian Fed.	≥1	≥1	≥1
Greece	≥1	≥1	≥1
Iran	≥1	≥1	≥1

**Table 2:** Description of the sample (*n* = 20)

**Country of birth and training/specialty**			
	Internal medicine	Psychiatry	Surgery
Romania	3	2	1
Poland	2	2	1
Russian Fed.	2	1	1
Greece	1	1	0
Libya	1	0	0
Iran	0	1	0
Syria	0	0	1
**Gender**			
Female			12
Male			8
**Years practised in Germany (at time of interview)**			
>10 years			7
5–10 years			7
1–5 years			3
<1 year			3

### Data collection

CK (female researcher in medical ethics with no prior relation to participants) conducted 20 face-to-face interviews in a setting chosen by the participants (8 in hospital ward, 8 in public space, 4 at home). The interview language was German. The interviews lasted between 20 and 80 min (mean 54 min) and were conducted between April 2013 and January 2014. Notes were taken afterwards on the recruitment process, set-up of interview, course of and possible disruptions to conversation, and discussions after the interview to aid later interpretations of data. Interviews were recorded and transcribed verbatim in accordance with the simplified transcription rules of Dresing and Pehl [54] by a professional transcription service. Transcriptions were controlled and anonymized by CK.

### Data analysis

The data were analysed by CK using qualitative content analysis adapted from Schreier [55]. A coding frame presenting the diverse difficulties was developed inductively from the data using the strategies of progressively summarizing and subsumption. The first 10 interviews were consensually coded [56] on a case-by-case basis in collaboration with a research assistant (a female advanced bachelor student of sociology) to ensure validity of data interpretation. A preliminary coding frame was constructed by comparing codes across interviews (progressively summarizing). Relevant passages of the next 10 interviews were compared against the coding frame and new categories created where necessary (subsumption). The resulting coding frame was discussed with other qualitative researchers to ensure its consistency and face validity.^7^ The coding frame was then checked against the original material by re-coding all interviews using MAXQDA-11. Relevant passages were translated into English for this article by CK.

## Results

In total, 20 interviews were conducted. The sample is described in Table 2. Participants described various difficulties relating to (a) the organization of healthcare institutions, (b) their own competencies, and (c) interpersonal interactions. Details of the described difficulties are given below accompanied by illustrative quotes.

### Experienced difficulties with the organization of the healthcare system

Participants experienced the organization of the healthcare system in many regards as deficient (see Table 3 for full overview). (Legal) norms regulating medical practice were seen as inadequate for various reasons. Participants criticized, for example, the obligation of extensive documentation as a waste of time. They also criticized the distribution of tasks between doctors and nurses as inefficient:

In Germany, doctors take blood and insert IV cannulas. In [country of origin] it is different. In [country of origin] nurses take blood. […] You need 1.5 hours of your working day for this. […] And I was always a little frustrated (laughs) because my colleagues working in France or Belgium tell me that over there nurses are still attending to those tasks. (Interview 3)

**Table 3:** Experienced difficulties with the organization of healthcare institutions

Inadequate norms regulating medical practice	Rules for treatment of psychiatric patients useless, harmful, coercive, and ambiguous
	Allocation of tasks between professional groups inefficient and unsatisfactory
	Excessive patient education and documentation of decisions
	Ineffective and too comprehensive specialty training
	Working/changing jobs burdensome with temporary licensure
Inadequate organization of routes for getting and preparing for a job as hospital physician	Licensure process slow and cumbersome
	Licensure requirements ineffective in securing competency
	Licensure requirements for non-EU applicants unfair and overburdening
	Future employer does not truthfully inform about working environment
	Inappropriate support to prepare for clinical work
Inadequate organization of hospital work and learning environment	No financial support, job prospects, and interesting tasks for interns
	Insufficient support for reaching postgraduate (specialist) training objectives
	Tasks assigned do not match level of expertise
	Inappropriate support to deal with difficulties experienced
	Staff shortages and excessive workload
	Further hospital-/person-specific inadequacies in organization
Further institutional difficulties	Career advancement difficult for women
	Important information not shared between different institutions
	Unfair differences in treatment of migrant doctors across time

Involving patients in decision-making was another topic addressed:

In our country, physicians were regarded differently. When a doctor says something, one has to listen. […] People listened; there was no need to have hour-long discussions with them. […] You could take the time for patients, for their clinical picture, for procedures, you see? And that was, as said, not so, stressful is not the right word, but you didn’t have to protect yourself so much. (Interview 11)

Licensing for work in Germany was also discussed critically. Specifically, a rule that only allowed EU nationals full (and not just temporary) licensure was considered unfair. Furthermore, the whole process of becoming licensed was criticized for being slow, confusing, and overly bureaucratic:

I: “Where is the bottleneck with your temporary licensure?” P: “With the job center. Well, I registered there five weeks ago, but always needed to wait, have eight new documents, further documents. That isn’t easy. […] Waiting for a month costs too much of my money and time.” (Interview 17)

In addition, the application process for specific hospital positions was criticized as physicians felt their future employer had lied to them about working conditions, e.g. support was promised but never offered.

You need help. That is just the way it is. […] And they promise all those things and then such a situation arises. And then most of us still have to manage on their own. And life is like that. I guess that is okay, but they should not make all those promises. That is a little disappointing. (Interview 20)

Moreover, participants were dissatisfied with the organization of their working and learning environment. They mentioned a lack of support structures for helping with adjustment (e.g. a tutor/mentor to ask questions or granting an adequate period for familiarization) and for advancing in postgraduate (specialist) training, e.g. no structured training plan was handed to participants. One participant was not even informed about the hospital’s limited permission for advanced training and that it would be necessary to move to another facility to finish postgraduate training. Additionally, participants often perceived the tasks assigned as either too demanding or below their capacities, considering their skill level:

In my country […] I was head physician, I had my own office. And it was a bit difficult for me to work again as resident physician (Assistenzarzt). And I had to ask questions as if I did not know. My senior physician (Oberarzt) said, I had to ask him everything. (Interview 12)

Then after a month they said I had to take care of patients by myself. And we are speaking of 16 patients. At the beginning, I found it difficult to do that alone. (Interview 3)

### Experienced difficulties with own competencies

Participants described that when starting work in Germany, they did not have all the setting-specific competencies they considered necessary for successful clinical interactions (see Table 4). In most interviews, language was an important topic, but participants struggled with diverse aspects. Some had difficulties understanding everyday language, while others struggled with unknown medical acronyms or names of pharmaceuticals, and others with colloquial terms for medical conditions.

**Table 4:** Experienced difficulties with own (setting-specific) competencies

Insufficient language competencies	Knowledge of everyday language
	Knowledge of medical jargon and nomenclature
	Knowledge of colloquial terms for medical issues
	Written expression and use of grammar
	Understanding dialects
Insufficient knowledge of the German healthcare system	The scope of own and co-worker’s responsibilities
	Organization of healthcare outside of hospital setting
	Legal requirements regarding treatment of patients and patient data
	Care processes and technical equipment used within hospital
Insufficient clinical competencies	Clinical knowledge exceeding own specialist field
	Experience with certain diagnostic/therapeutic techniques
	Experience with treating certain medical conditions
	Further necessary practical skills like taking blood
Insufficient cultural knowledge	Cultural and historical context as basis for psychotherapy
	Cultural (implicit) rules of conduct like how to show emotions
Further competencies lacking	Knowledge of machines causing accidents as basis for surgical interventions
	Handling own deficiencies/insecurities well
	Adjusting to and accepting the new environment

Understanding the German healthcare system was another difficulty. Specific issues discussed ranged from technical details (e.g. computer system used for patient management) to learning that organizing psychosocial care for patients was part of the care team’s (especially the social worker’s) responsibility. Some important legal requirements were also unknown:

For example: If a relative calls the ward and wants to talk to a doctor. In [country of origin] you talk to them. It’s family. Here the patient needs to first sign a release from confidentiality and then you can talk to them. I did not know that at the beginning. I did talk to the partner. God bless, […] they did not want to sue me. (Interview 20)

Furthermore, some participants reflected on their clinical competencies. They lacked, for example, the necessary experience with treating certain diseases like tuberculosis because they were not common in their country of origin, or were assigned to another specialist group. The same problem arose in using certain diagnostic/therapeutic tools. Some struggled because they felt that, in Germany, they needed competencies exceeding their specialization:

Because compared to [country of origin] where you do a narrow, where I predominantly did cardiology and that was it, in Germany I had to care for all of internal medicine, also during night shifts. That was professionally challenging. And that is the same everywhere in Germany, I realized, also during night shifts. Although you are a cardiologist, you are responsible for the whole of internal medicine. That was difficult at the beginning. (Interview 3)

Psychiatrists in particular mentioned a lack of cultural knowledge with regard to implicit expectations of patients and colleagues:

Yeah, when I have an idea, an advice, I cannot really put it across to my patient. I am myself enthusiastic about the idea, but the patient looks at me… I get the feeling, this is all about, he either did not really understand me or it does not fit the nationality – could one say – or the culture. (Interview 9)

Further issues described relate mainly to meta-capacities of adjusting to the new system or dealing well with one’s own insecurities, e.g. that one finds it difficult to approach and ask senior physicians for help.

### Experienced difficulties in interpersonal interactions

Further difficulties relate to interpersonal interactions with patients, colleagues, and superiors (see Table 5). Often, participants felt badly treated by colleagues (including nurses) and patients. Experiences of rejection and discrimination were frequently attributed to their status as “foreigner”, although other attributes like gender were also important:

Concerning acceptance from colleagues, how do you say, rejection. Well, in fact, they wanted to prove at all costs that it (note CK: working in psychiatry) cannot be accomplished by a foreigner. [....] And well, constantly some insulting or, how can one say, remarks or jokes about [country of origin].[…] I resigned after three months. (Interview 9)

**Table 5:** Experienced difficulties in interpersonal relations and interactions

Inadequately treated by patients or co-workers	Rejected or discriminated as foreigner
	Rejected or discriminated because of other attributes
	One’s professional competencies are misjudged/mistrusted
	Annoyed, impatient, or unsupportive reactions to (language) deficiencies and questions
	Not appreciated for effort and achievements
Certain characteristics of patients or co-workers complicate teamwork/patient care	Co-worker of different opinion regarding adequate patient care
	Co-workers lack necessary competencies or motivation
	Hearing impaired/old patients’ inability of proper expression
Further difficulties in professional interactions/relations	Burdened by history of military conflict between countries of origin
	Family issues impair professional performance
	Diverse behaviour patterns and attitudes of people found irritating to infuriating

Equally difficult to handle for participants were negative, often annoyed reactions to questions which made it even harder for participants to compensate for limited setting-specific competencies. They also struggled with lack of trust in (sometimes also excessive expectations of) one’s professional competency:

I realized that I have to prove myself more. If I make a mistake, everybody thinks that I made it because I do not understand. A German doctor can make the same mistake, but then it is just a mistake. […] For example, I wanted to write 1.5, but the comma wasn’t well printed, it read 15. […] And they were thinking: She does not understand, she wanted to give 15 instead of 1.5. Small things, but frustrating. (Interview 20)

Another point of annoyance was when patients or co-workers lacked important competencies for adequate care. Younger doctors who had not yet acquired the necessary skills or lacked motivation—and thereby complicated effective teamwork—sometimes annoyed participants with advanced careers. Interestingly, participants were particularly critical of other migrant physicians.

Further difficulties described relate, for example, to behaviour of patients and colleagues which had been perceived as inappropriate. Sometimes the perceived inadequacy in behaviour was attributed to cultural differences. One participant found it very hurtful that nobody ever invited her to have breakfast with the rest of the team. Her explanation for this inconsiderate behaviour was the limited importance the German culture attaches to hospitality. However, this attribution was not always made:

Back then I was working with a head physician, he well, how shall one say, was a narcissist. He screamed at all of us, we were all stupid – German or foreign – all were equally (laughs) bad. (Interview 19)

Another interesting point was raised with regard to former military conflict between home countries that complicated physician-patient interactions:

A nice grandpa, 90 years old who came with three…, well, who ended up with me. I was the physician responsible for his ward. For three days he did not want to talk one word with me. On the third day, he started crying and then I asked him, why, what the issue was. And he said that he was at the Eastern front and he shot [citizens of country of origin]. He never thought that someone from [country of origin] would ever treat him. That was very pleasant (ironic). (Interview 7)

## Discussion

To our knowledge, this is the first study on difficulties experienced by migrant physicians in German hospitals demonstrating the broad spectrum of issues this group might struggle with. Several difficulties identified in our study have also been described in studies from other contexts [8–29] while some, to our knowledge, have never been described before and might possibly be specific to the German context; however, this would need further analyses. Former military conflict between the countries of origin burdening the physician-patient relationship or frustration with work distribution between nurses and doctor are examples of such difficulties.

Interestingly, the issue of inadequate organization of the hospital as a working and learning environment has only been highlighted by two further studies [26, 28, 29]. The explanation for the prevailing silence on this issue is probably not that it does not arise in other (national) settings. More likely is that authors presume that this issue is not specific to migrant physicians and therefore not worth mentioning—as explicitly discussed in Chen et al. [21]. Although we do agree that, e.g. the issue of lacking support during postgraduate training is not specific for migrants [57, 58], we do not see good reasons to judge any described difficulty as migrant-specific. Due to the lack of similar data from other groups, we think those exclusion decisions are highly questionable and possibly represent underlying stereotypes of researchers. In fact, most of the described issues may also be experienced by German-trained physicians (possibly less severe). Some might actually be typical for *any* physician starting residency training (e.g. colleagues being annoyed by questions) emphasizing the need to implement better support structures in general.

As mentioned before, the described difficulties can negatively affect participants’ job satisfaction and might thereby indirectly affect retention [28, 33] and quality of patient care [32]. Some issues might also directly affect the quality of patient care, e.g. where language difficulties cause misunderstandings and consequently mistreatment. Participants of our study also recounted episodes of depression and change of employers or even specialities due to perceived difficulties. In light of these possible consequences, policymakers should not take those issues lightly but devise strategies to explicitly address them. Due to limited space, we will focus our discussion on four issues and the corresponding possibilities for policy action we consider specifically relevant: (1) misinformation and further malpractice in the recruitment process, (2) experiences of discrimination, (3) unease with certain norms and behaviour, and (4) limited setting-specific knowledge.

Difficulties seem to begin in the recruitment process. In our study, and in another conducted in Ireland [28], participants complained about practices of misinformation. Misinformation and other questionable practices (e.g. excessively high breach-of-contract fees) in recruitment have received considerable attention in the context of nurse migration [59]. However, much less is known about the recruitment procedures (e.g. how do physicians find jobs) and actors involved (e.g. importance of private recruitment agencies) when it comes to migrant physicians. Further research into the recruitment of foreign-trained physicians would be helpful for devising adequate policy responses. However, deceiving future employees about working conditions is already illegal in the German context and can have ramifications for institutions involved,^8^ which is generally a strong incentive to refrain from dubious practices. Therefore, what can be done by policymakers to motivate change in information practices of healthcare institutions is not straightforward, except to ensure migrant physicians are informed about their legal rights. In the United States, the Voluntary Code of Ethical Conduct for the Recruitment of Foreign-Educated Health Professionals to the United States was developed and implemented by various stakeholders to achieve such an objective [60]. However, it is questionable whether voluntary codes can be effective in incentivizing change. Their success will probably depend to some extent on visibility and monitoring/reporting capacity [61]. If, however, such a code shows to be effective, it will also be helpful in motivating institutions involved to address other issues discussed below.

Discrimination of foreign-trained physicians (not necessarily as migrants but also, for example, as women) is another important issue according to our interviews. Discrimination experienced by participants mostly took the form of insulting remarks with regard to ethnicity/nationality by colleagues, which even led one participant to resign after just 3 months. Severe forms of this kind of harassment are also illegal in Germany, and hospitals have a legal obligation to protect employees from it [62], e.g. by dismissing the offenders. However, most discriminatory acts will likely be more subtle and therefore not justiciable. Healthcare institutions should also address these conflicts to maintain a satisfied and motivated workforce. Creating a welcoming atmosphere and fostering an understanding of diversity as an opportunity, instead of (just) a challenge, is clearly a leadership task for healthcare institutions. Management strategies could be adopted from the business sector where diverse workforces and clients have long been the norm and the necessity for actively managing diversity has been acknowledged [63]. Effective strategies for healthcare institutions have been identified (e.g. including respect for diversity as a goal in the mission statement, diversity awareness, and conflict management trainings) but insufficiently taken up by decision-makers in healthcare institutions in Germany [64, 65]. At the policy level, similar strategies as described before—informing migrant physicians about legal rights and possibly implementation of a code on ethical conduct—should be considered to motivate change at the institutional level.

Furthermore, participants considered certain norms and behaviour—often practice norms that were notably different from what they were used to in countries of training—as inadequate. This issue has been indicated in other publications [11, 15, 22, 23] but mostly conceptualized as educational need. Our findings, however, demonstrate incongruence between physicians’ attitudes and (moral) values and those imbedded in certain structures or behaviour. This causes frustration and dissatisfaction, which cannot be overcome by educational interventions because it does not arise from a lack of understanding. Although we cannot offer ready-made solutions to this issue, we want to propose routes for further thought. First, irritation of this kind can be an opportunity: the physician brings to the hospital intimate knowledge of another healthcare system, which can serve as a benchmark for the system she is currently working in. Her irritation can be a first step in critically reflecting and possibly improving structures. Based on our findings, for example, policymakers or hospital managers may want to reconsider whether certain tasks that are executed by physicians could be taken over by nurses, thereby possibly improving efficiency and job satisfaction of both professional groups [34, 66]. Second, identifying strategies to deal appropriately with frustration caused by diverging attitudes and tastes will require management and psychological insight. However, where the points of contention are moral values, the situation becomes more complicated^9^ as there are no universally accepted methods to resolve these normative conflicts. So far, the focus in the literature has mostly been on difficult situations involving migrant patients and various approaches for resolving conflict have been discussed (e.g. culturally sensitive ethics consultations [67, 68]). The intricacies of value conflicts between migrant physicians and their patients, colleagues, or the system as such—posing different problems due to the differences in power dynamics—are so far largely ignored. Further investigations by normative scholars would be desirable for the future.

In addition, participants expressed that limited setting-specific competencies posed difficulties for them. Participants mentioned inadequate language abilities but also insufficient system, cultural, and clinical knowledge due to the differences in healthcare systems and societies more generally. So far, mainly language deficiencies have been on the political agenda in Germany with the conference of ministers of health aiming at harmonizing language testing across states and tailoring it to vocabulary needed in medical settings [69]. However, it is not enough to require language testing; adequate language courses must be offered allowing migrant physicians to acquire relevant competencies. Otherwise, language testing might just become an insurmountable barrier to licensure resulting in brain waste or onward migration. Our findings have furthermore demonstrated a much broader need for support than just language courses. Strategies addressing these needs have been developed and positively evaluated in the international literature. A systematic review published in 2015 [70] identified 22 interventions addressing diverse learning needs and using various methods like presentation and discussion, mentoring, individual study activities, simulation, and role-play. Participants of an Australian study have identified additional forms of information provision (booklets or web-based) as helpful [71]. However, the methodological rigour of studies was low, and additional research needs to be conducted to demonstrate effectiveness of support programmes and transferability to other contexts. So far, there exists no mechanism in Germany to ensure that all migrant doctors receive appropriate support if needed, although a few private initiatives offer comprehensive training programmes [34].^10^ It is, furthermore, not clear who should be considered responsible for implementing support structures and who should be held accountable if mistakes happen due to a lack of support. Irrespective of how these questions get answered, policymakers need to ensure, via regulation, that training programmes are offered nationwide.

We simultaneously want to caution against (over-) generalizing our findings as support needs will differ significantly between physicians—an observation we could also make for our sample. When extensive system knowledge has been acquired, for example, during previous internships, obligatory orientation training is unnecessary and possibly offensive if it reflects a negative stereotype. As demonstrated by the second last quote in the “Results” section, being pre-judged based on stereotypes related to one’s competencies can be a painful experience. Accordingly, one-size-fits-all approaches cannot be an adequate solution, but programmes have to be adaptive and flexible to individual physicians and their needs (while being prepared to address typical issues). Most likely, physicians trained in Germany will also benefit from these support strategies.

### Limitations

First, we did not investigate a representative sample, and therefore, our study results cannot be generalized to other migrant doctors. Neither does the study allow conclusions to be drawn about differences between subgroups or the severity of the identified issues. It can, however, serve as the necessary starting point for further quantitative research projects.

Another limitation may have been that the interviewer was a young (27 years), female researcher without a medical background. However, in all interviews, a positive and trustful atmosphere could be created so that participants felt comfortable to share their stories. Although all participants had sufficient German language skills, the interview language (German) might have caused misunderstandings. There is a risk that the focus of the study on difficulties might have led participants to overstate their experiences or opinions; however, they might have also downplayed their experiences as problems and mistakes are still a taboo in medicine. Furthermore, stories shared might have suffered from recollection bias. In addition, some difficulties experienced will no longer be of policy relevance due to institutional and political changes.^11^

## Conclusions

Migrant physicians might face diverse difficulties upon practising in Germany that should be addressed on various levels to ensure physician wellbeing, retention, and quality of care. Assuming that the identified problems do not just reflect specific individual experiences, different strategies to support migrant physicians in acquiring setting-specific knowledge should be developed for the German context, evaluated, and offered on a large scale while being sensitive to migrant physicians’ varying needs for support. Furthermore, discrimination of “foreign” physicians calls for better diversity management in hospitals. To retain (migrant) physicians, it will be equally important to improve further organizational deficiencies. On the policy level, a code on ethical conduct might be one possible strategy to motivate institutional change—especially, but not exclusively, regarding questionable recruitment practices. Further research should establish the frequency and severity of the described issues and their possible consequences and look into recruitment practices. Additionally, normative-analytical research should address questions of allocating responsibility for implementation of support structures and how to deal with divergent (moral) values in the healthcare system.

## Footnotes

1 Among them are the following countries: USA, Canada, Australia, UK, Ireland, Israel, Austria, Belgium, Sweden, and Norway.

2 In most healthcare systems, postgraduate training is required for full (specialist) licensure which allows unsupervised practice. Depending on the system, migrant physicians not able to enter a training programme might not be able to work clinically at all or end up working in a non-training post with limited career opportunities.

3 “Foreign” should be understood as foreign-trained in accordance with the OECD report. Germany, however, does not collect data on foreign-trained physicians but defines “foreign physician” by nationality (Staatsbürgerschaft) which weakens comparability. Following the OECD, we use this data as the closest estimate of the number of foreign-trained physicians in Germany.

4 Data from 2014 (Norway, Ireland, UK) and 2012 (Switzerland).

5 Directive 2005/36/EC

6 Obviously, there are further factors influencing experiences of migrant physicians (e.g. age). However, for pragmatic reasons, we had to limit the factors we varied to two.

7 This concept is concerned with whether something appears (but cannot or has not been proven) to be appropriate with regard to its goals.

8 Under certain conditions, employers might have to compensate for harms accrued due to conclusion of the contract (according to §§ 280 I, 311 II Nr. 1, 241 II BGB, see also BGH, NJW-RR 1997, 144; Palandt/*Grüneberg*, § 311 Rn. 40).

9 Although value conflicts are not specific to intercultural (physician-patient) encounters, those interactions might be of intensified conflict.

10 One example would be the VIA Institute for Education and Profession in Nuremberg (http://www.via-institut.de/).

11 The 2012 recognition law (Anerkennungsgesetz) has, for example, brought significant changes to licensure procedures and thereby resolved some issues discussed. Non-EU nationals, for instance, are no longer excluded from full licensure.
